# Semiautomatic Cochleostomy Target and Insertion Trajectory Planning for Minimally Invasive Cochlear Implantation

**DOI:** 10.1155/2014/596498

**Published:** 2014-07-02

**Authors:** Wilhelm Wimmer, Frederic Venail, Tom Williamson, Mohamed Akkari, Nicolas Gerber, Stefan Weber, Marco Caversaccio, Alain Uziel, Brett Bell

**Affiliations:** ^1^ARTORG Center for Biomedical Engineering Research, University of Bern, 3010 Bern, Switzerland; ^2^Department of ENT, Head and Neck Surgery, Inselspital, University of Bern, 3010 Bern, Switzerland; ^3^Otology and Neurotology Department, University Hospital of Montpellier, 34961 Montpellier, France; ^4^Institute for Neurosciences of Montpellier, INSERM U1051, 34091 Montpellier, France

## Abstract

A major component of minimally invasive cochlear implantation is atraumatic scala tympani (ST) placement of the electrode array. This work reports on a semiautomatic planning paradigm that uses anatomical landmarks and cochlear surface models for cochleostomy target and insertion trajectory computation. The method was validated in a human whole head cadaver model (*n* = 10 ears). Cochleostomy targets were generated from an automated script and used for consecutive planning of a direct cochlear access (DCA) drill trajectory from the mastoid surface to the inner ear. An image-guided robotic system was used to perform both, DCA and cochleostomy drilling. Nine of 10 implanted specimens showed complete ST placement. One case of scala vestibuli insertion occurred due to a registration/drilling error of 0.79 mm. The presented approach indicates that a safe cochleostomy target and insertion trajectory can be planned using conventional clinical imaging modalities, which lack sufficient resolution to identify the basilar membrane.

## 1. Introduction

The aims of minimally invasive cochlear implant (CI) surgery are manifold. On the one hand, minimally invasive access to the cochlea is gained through a direct cochlear access (DCA), which is a small tunnel drilled from the mastoid surface to the cochlea passing through the facial recess [1, 2]. In addition to a minimally invasive access, the preservation of intracochlear structures during and after electrode array insertion is an important research topic.

Once access to the tympanic cavity is established, the cochlea must be opened to enable CI electrode array insertion. Two criteria are primarily considered in the current definition of atraumatic electrode insertion. First, the scala tympani (ST) is the favored intracochlear lumen for implant placement, especially in terms of retaining residual hearing [3–7]. Second, the ideal insertion trajectory should align with the center line of the ST to prevent damage to the basilar membrane, the modiolus, or the spiral ligament during insertion. The ST can be accessed either through a strict round window (RW) approach, a RW related cochleostomy, or a promontory cochleostomy separated from the RW. Drilling the cochleostomy in the correct location is one of the major challenges the surgeon faces during the surgery. The position is chosen intraoperatively according to the anatomical situation of the promontory (i.e., inferior or anteroinferior to the RW membrane) to avoid damage to basal intracochlear structures [8–14].

In this context, image-guided cochleostomy approaches have been investigated to aid the surgeon in determining the proper drill site, but, to our knowledge, no clinical data has been published [15, 16]. Correct planning of the cochleostomy site and insertion trajectory rely on an accurate representation of the anatomy during planning. However, clinically applicable imaging modalities do not provide sufficient imaging resolution for direct detection of the ST. The RW remains the only consistent anatomical landmark for preoperative/intraoperative ST access planning. Meshik et al. analyzed insertion trajectories in cadaveric temporal bones using microcomputed tomography (micro-CT) imaging for ST visualization and subsequent centerline computation [17]. An alternative approach utilizes active shape modeling for approximation of the position of the ST. The first report of a clinical implementation of this concept showed complete ST implantation in 6 of 8 patients with minor complications [2].

Anatomical variations play an important role in the planning and execution of any surgical procedure. For this reason, we hypothesize that an interactive method is most appropriate during the planning phase as this leaves the ultimate decision in the hands of the surgeon and avoids errors arising from automatic “black box” methods. Furthermore, we posit that the accuracy afforded by an image-guided robotic system can allow the surgeon to perform the cochleostomy with sufficient accuracy to reliably place the electrode within the ST. This work will present a semiautomatic planning method, which allows the user to plan the cochleostomy site and insertion trajectory compared to an idealized centerline approach [17]. The method was tested in a whole head cadaver model wherein the planned trajectory and cochleostomy site were drilled using an image-guided robot system.

## 2. Materials and Methods

### 2.1. Cochleostomy Position and Insertion Trajectory Computation

To obtain cochleostomy target positions and insertion trajectories, a semiautomatic landmark based approach was implemented. The method consists of three subsequences: manual landmark identification, surface model generation of the cochlea, and automatic cochleostomy and trajectory computation.

**Figure 1:**
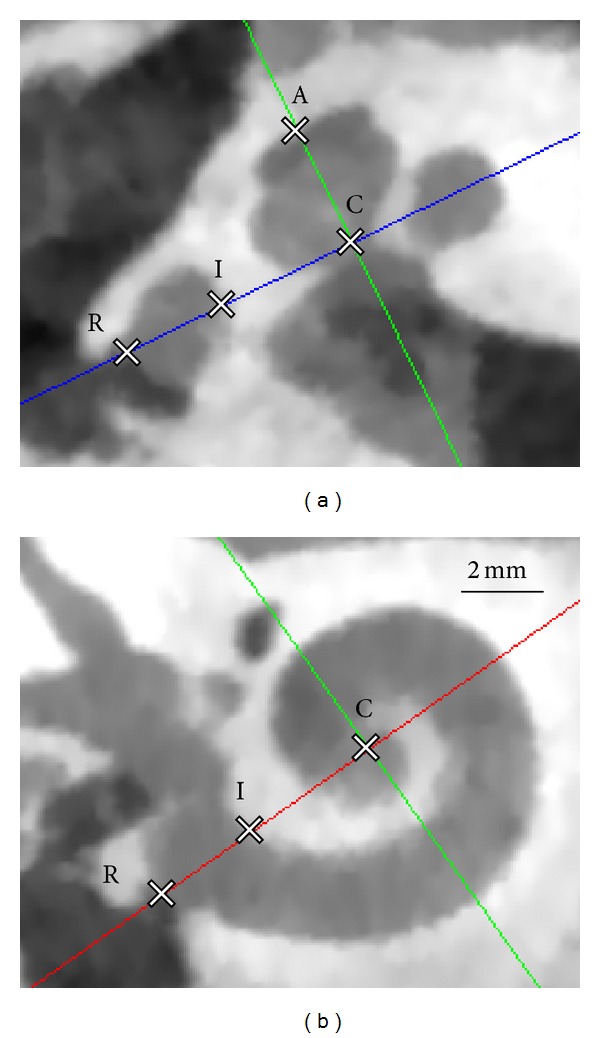
Landmark identification of a right human cochlea using cone beam CT data. (a) Oblique axial slice corresponding to the 0° reference plane (red line in (b), as defined in [18]). The RW center adjacent to the bony overhang (R), the inner wall border at the RW (I), the center of the modiolus in the basal turn (C), and the apical center of the modiolus (A) are used to define a local cochlear coordinate system for further computations. (b) Oblique coronal slice of the basal turn (blue line in a) and corresponding in-plane landmark positions.

Landmark identification and cochlear surface model generation were performed in a medical image analysis software (Amira 5, VSG, Burlington, MA, USA). Oblique coronal, axial, and sagittal slices were aligned to visualize the cochlea according to international consensus [18]. As landmarks, the center of the RW at the bony overhang (R), the basal center of the modiolus (C), the apical center of the modiolus (A), and the inner wall at 0° reference angle (I) were defined (Figure 1). Further, the cochlea, the vestibulum, and the semicircular canals were segmented using a region growing algorithm. Structure labels outside the bony labyrinth were manually removed and a three-dimensional surface model was generated.

**Figure 2:**
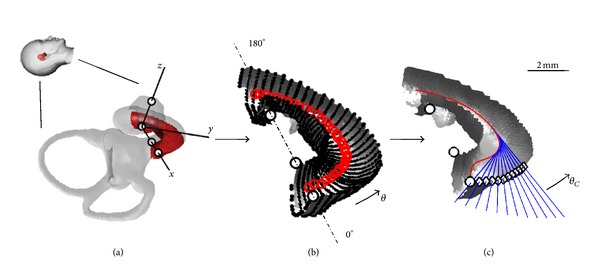
Illustration of the automatic cochleostomy target/insertion trajectory computation algorithm. (a) Based on the landmarks (black circles), a local cochlear coordinate system is computed. As an assumption, the *x-y* plane is defined as the location of the basilar membrane. The surface model of the cochlea is truncated to the first half turn of the ST. (b) Radial cross sections are computed starting at the RW (0° reference). The center of gravity is estimated (red circles) based on the extracted vertices (black dots) for each cross section. (c) The centroid line (red line) is fitted with the data points, representing the mid-scala course of ST. For a specified range, the tangents of the centroid line are computed, defining the optimal insertion trajectories (blue lines) and the corresponding cochleostomy targets (diamonds) at the angular position *θ*_*C*_.

A Matlab script (The MathWorks Inc., Natick, MA, US) was implemented for automatic cochleostomy target and insertion trajectory computation. The coordinates of the previously found landmarks and the cochlear surface model serve as input for the algorithm. A local cochlear coordinate system based on cochlear landmarks is created (Figure 2(a)). The origin of the coordinate system is placed in the basal center of the modiolus (C). The *x*-axis runs through the RW (landmark R), and the *z-*axis passes through the apical center of the modiolus (A). Finally, the *y*-axis is computed as the cross product of *x* and *z*. The cochlear model is simplified through two assumptions. First, the location of the basilar membrane is assumed to lie in the *x-y* plane in the basal turn of the cochlear model. Close to the RW membrane this assumption may not apply, since the basilar membrane orientates along the *x-z* plane [11]. Nevertheless, the simplification ensures that insertion trajectories are not oriented toward the basilar membrane in the basal turn. Additionally, it is supposed that the basilar membrane is not lying posterior to the *x-y* plane (i.e., negative *z* coordinates). The second major assumption is that the width of the ST in the region of interest does not exceed the distance between the landmarks R and I. The first stage of the algorithm involves the identification of surface points belonging to the ST. This is performed by truncating the set of points to those having positive *y* and negative *z* coordinates (Figure 2(a)). Next, the algorithm removes points not belonging to the basal ST surface by satisfying the assumption that the ST width is no larger than the distance between R and I. The third step of the algorithm is the extraction of ST radial cross sections which are used to compute the ST centerline. This is accomplished by finding the nearest neighbor of a plane coincident with the *z-*axis with discrete angular steps (i.e., Δ*θ* = 5°; see Figure 2(b)), starting at the RW (*θ* = 0°) and extending throughout the first basal half turn (*θ* = 180°). The center of gravity is calculated from the surface points in each cross section. Finally, a cubic spline is fit to the centers of gravity to approximate the mid-scala course of ST. An optimal insertion trajectory is defined as a line tangent to the smoothed spline at a defined basal turn angle *θ*. The corresponding cochleostomy points are found using a ray/triangle intersection algorithm [19]. The insertion trajectories and target points are computed in steps of 2° up to a maximum of *θ*_*C*_ = 20° (Figure 2(c)).

### 2.2. Basilar Membrane Approximation Error

**Figure 3:**
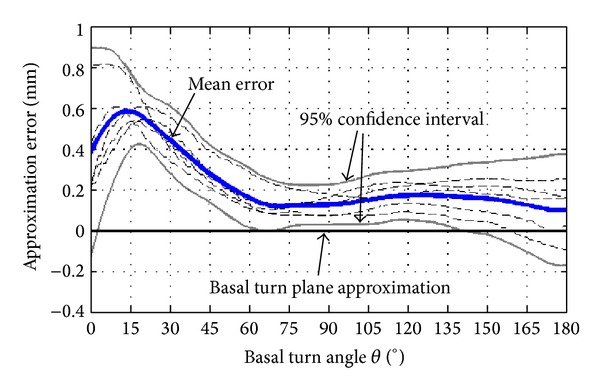
The distance between the approximated position of the basilar membrane (as computed with the landmark based approach) and its actual position in the corresponding micro-CT data (blue line) of 5 human cochleae is shown. An average error of 0.22 mm was observed in the region used for insertion trajectory computation (60° ≥ *θ* ≥ 45°).

In order to verify that the assumptions for the approximation of the basilar membrane location apply, five datasets consisting of cone-beam CT and micro-CT images of human cochleae were used. Images of both modalities were registered and the displacement error between the actual position of the basilar membrane (micro-CT) and the approximated location (*x-y* plane, as found with the landmark based approach in cone-beam CT) was assessed. An overall mean error of 0.23 mm was found for the first half of the basal turn. As expected, the error is higher close to the RW. In the region used for trajectory computation (60° ≥ *θ* ≥ 45°), an average error of 0.22 mm was measured (Figure 3).

### 2.3. Ex Vivo Validation Study 

#### 2.3.1. Specimen Preparation and Preoperative Imaging

Five human cadaver heads (*n* = 10 temporal bones) fixed with 20% zinc chloride intra-arterial injection were used in this study. A minimally invasive access to the tympanic cavity was drilled with a purpose-built robotic system developed in Bern [1]. The system uses bone-anchored fiducial titanium screws for patient-to-image registration [20]. All experimental parts of the study (i.e., intervention planning, drilling, and array insertion) were performed in a laboratory of the University Hospital of Montpellier, France. High resolution cone-beam CT scans (NewTom 5G, QR S.r.l, Verona, Italy) were acquired (voxel size: 125 *μ*m isotropic, 110 kVp, 19 mA). For intraoperative endoscopic examination of the surgical procedure through the external auditory canal, the tympanic membrane was removed in all specimens.

#### 2.3.2. Surgical Intervention Planning

**Figure 4:**
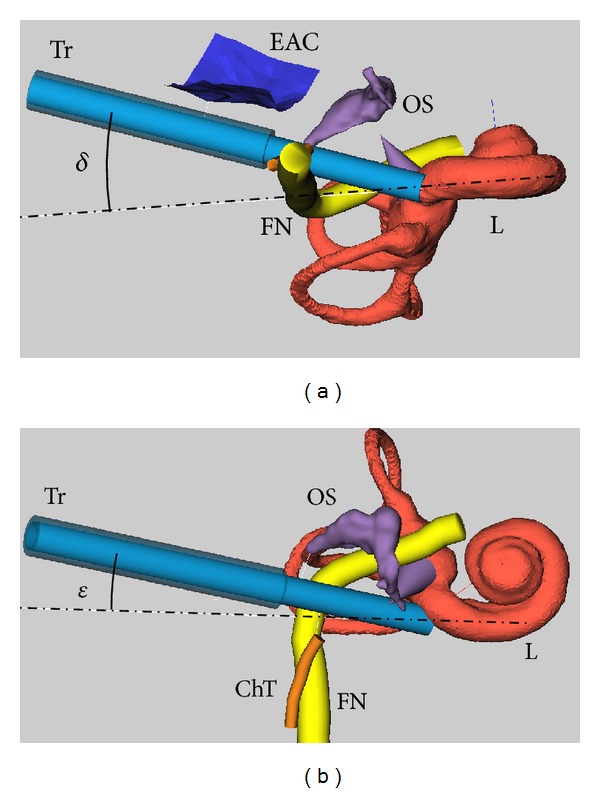
Intervention planning for minimally invasive CI surgery in a dedicated software tool [21]. Visualization of the segmented posterior wall of the external auditory canal (EAC), the facial nerve (FN), the chorda tympani (ChT), the ossicles (OS), and the bony labyrinth (L). The planned trajectory (Tr) and the ideal trajectory as computed by the algorithm (broken-dotted line) are shown. (a) Planning situation from an inferior view; the angle *δ* describes the offset between the planned trajectory and the ideal trajectory with respect to the basal turn of the cochlea for a given cochleostomy target. Note that the ideal trajectory is running through the facial nerve. (b) The same plan as seen from an anterior view; the offset between the planned and the computed ideal trajectory in the basal turn plane is described by the angle *ε*.

The computed cochleostomy targets and trajectories, as well as the surface model of the cochlea, were imported into a dedicated surgical planning software [21]. The software allows the user to manually choose the drill/insertion trajectory based on the distances to critical structures in the temporal bone (i.e., facial nerve, chorda tympani, posterior wall of the external auditory canal, and the ossicles) and in relation to the computed ideal trajectory. In practice, the user defines a cochleostomy site (*θ*_*C*_) and then adjusts the drill trajectory to minimize the deviation from the ideal. Two angular measures were introduced to facilitate this process [22]. First, the out of plane component is described by the angle *δ*. Second, the in-plane alignment is given by the angle *ε* as seen in Figure 4. Negative *δ* and *ε* values should be avoided as this indicates a collision with the basilar membrane and the modiolus, respectively. The final plan and alignment of the trajectory were performed by an experienced ENT surgeon with the goal of minimizing *δ* and *ε*.

#### 2.3.3. DCA Drilling and Cochleostomy

The DCA tunnel was drilled using the same protocol published previously [1]. The DCA was drilled by the robot using a custom “step” drill having a proximal diameter of 2.5 mm with a length of 20 mm and distal portion with a diameter of 1.8 mm and a length of 10 mm to the tip. The drill motor was started (5,000 rpm) and the robot drilled with a feed rate of 0.5 mm/s using a “pecking” motion until the middle ear cavity was reached. A cochleostomy was then drilled (1 mm diamond burr) using the robot system. The drill speed was increased to 10,000 rpm, and the feed rate was reduced to 0.1 mm/s.

#### 2.3.4. Electrode Array Insertion

Electrode array insertion was performed by two experienced ENT surgeons using the same protocol. Ten free-fitting electrode arrays (Med-El Flex^28^, 28 mm array length) were used for the experiments. The DCA tunnel was cleaned using irrigation and aspiration via the external auditory canal. Hyaluronic acid was injected into a custom insertion tool (which provides alignment to the cochleostomy) for lubrication. Next, the electrode arrays were carefully straightened and slowly introduced into the tool lumen and the progression into the cochlea was observed with a 4 mm 30° endoscope through the external auditory canal. Advancement of the electrode array was stopped at the point of first resistance. After completion of insertion, electrode arrays were fixed using sutures to prohibit movement during subsequent handling phases. During the experiments, the insertion time and tactile feedback of the insertion were recorded.

#### 2.3.5. Postoperative Imaging and Data Analysis

Postoperative scans were acquired using the same protocol as in the preoperative phase with and without the implanted electrode arrays. The pre/postoperative datasets were registered by aligning the surfaces of the implanted fiducial screws (Amira 5). The accuracy of the drilled DCA tunnel was assessed by comparing the segmented tunnel position with the planned trajectory as previously reported [1]. The drilled trajectory target error, alignment (angles *δ* and *ε*), the actual cochleostomy position (*θ*_*C*_), the implanted scala, the angular insertion depth, and the number of intracochlear contacts were assessed. Furthermore, three-dimensional visualizations were generated for additional evaluation.

## 3. Results

### 3.1. Cochleostomy Target/Trajectory Computation and Planning

Preoperative imaging resolution and quality were sufficient for identification of the specified landmarks and for segmentation of the bony labyrinth. The presented script generated cochleostomy targets at positions inferior to the RW membrane. Visual inspection of image data showed effective alignment of the computed trajectories with the basal turn. Preprocessing, including landmark identification, bony labyrinth segmentation, and computation of cochleostomy targets and trajectories, took approximately 15 min on average for each case. In all cases, the output of the script was used for subsequent trajectory planning. Due to a narrow facial recess, it was planned to sacrifice the chorda tympani in three cases (see Table 1).

### 3.2. DCA Drilling and Cochleostomy

**Figure 5:**
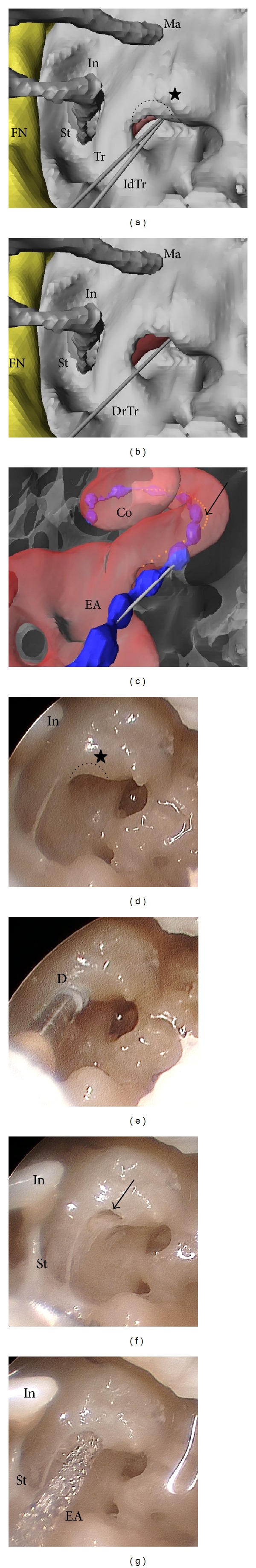
Three-dimensional virtual view of the promontory (a)–(c) and corresponding endoscopic photo documentation (d)–(g) during cochleostomy drilling and array insertion in specimen 2R. The facial nerve (FN), the stapes (St), the long process of the incus (In), and the malleus (Ma) provide orientation landmarks. (a) The planned trajectory (Tr) and the computed ideal trajectory (IdTr) are shown. The cochleostomy (dotted semicircle) is aimed at drilling through the RW bony overhang (black star). (b) View of the promontory after cochleostomy with corresponding drilled trajectory (DrTr). (c) Transparent view of the promontory after insertion of the electrode array (EA). The cochlea (Co) and the centroid line as computed by the algorithm (arrow) are shown. (d) Promontory prior to cochleostomy drilling (dotted semicircle) at the RW bony overhang (black star). (e) Cochleostomy drilling with a 1 mm diamond burr (D). (f) Promontory with cochleostomy (arrow). (g) After insertion of the electrode array (EA).

Robotic DCA tunnel and cochleostomy drilling were feasible in every case (Figure 5(e)). The accuracy at the cochleostomy target was measured at 0.30 ± 0.23 mm with a range of 0.05 to 0.79 mm. Four cases had broken screws which likely caused some degree of error in the registration process. In two of these cases a target error bigger than 0.35 mm occurred (Table 2). The target error was orientated anteriorly and posteriorly in specimen 1L and 1R, respectively. This caused penetration of the external auditory canal posterior bony wall in specimen 1L and a close passage of the facial nerve in specimen 1R. As expected, the chorda tympani was damaged in specimens 2L and 2R.

### 3.3. Electrode Array Insertion

**Figure 6:**
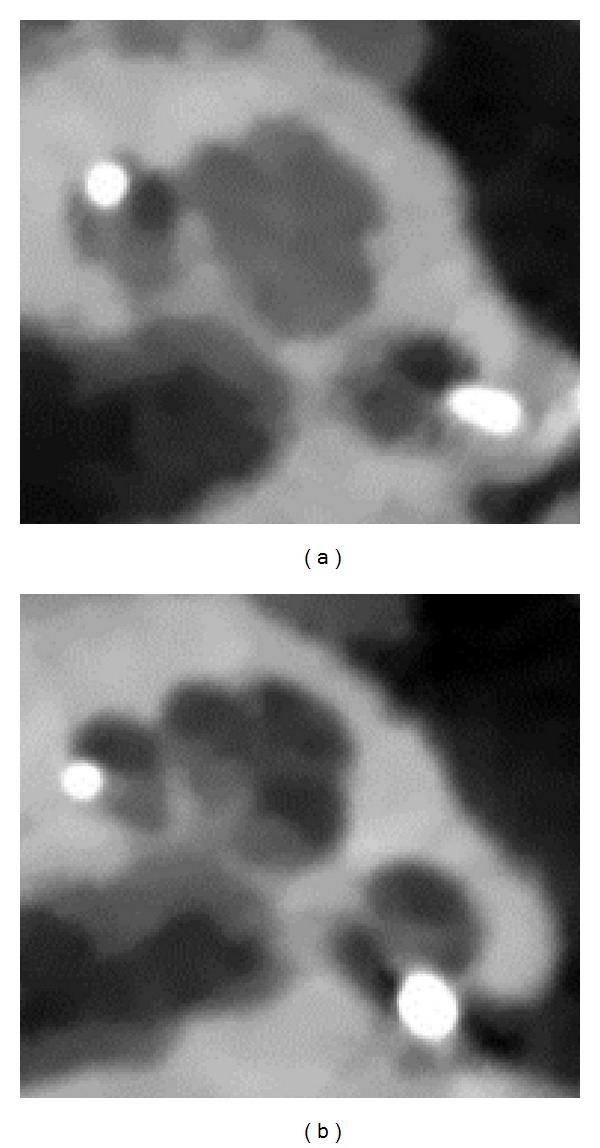
Radiological evaluation of the insertion outcome in axial cone beam computed tomography slices. (a) Left cochlea with scala vestibuli insertion caused by a target drilling error of 0.79 mm orientated anteriorly (specimen 1L). (b) Complete ST insertion in a left cochlea (specimen 4L).

Endoscopic examination demonstrated correct alignment of the drilled DCA tunnel and insertion tool with the cochleostomy (Figure 5(f)). Manual electrode array insertion was feasible in all cases (Figure 5(g)). Full insertion as indicated by the mark on the electrode array was achieved in 2 of 10 cases with an average angular insertion depth of 319° (Table 2). The total insertion procedure took 5 min on average. Postoperative radiological examination showed 9 of 10 cases of complete placement into ST and 1 case of scala vestibuli insertion caused by a drilling target error of 0.79 mm (Figure 6).

## 4. Discussion

This study investigates the applicability of a landmark-based algorithm for patient-specific cochleostomy target and insertion trajectory computation. In the presented method, the lack of visualization of intracochlear structures in clinical computed tomography images is compensated for by the assumption that the basilar membrane position can be approximated based on specific landmark positions. These landmarks are easily identified and are based on a recognized scheme for cochlear visualization [3, 10, 18, 23, 24]. Furthermore, the landmarks enable the straight forward creation of a local cochlear coordinate system which has utility in the described planning method, as well as for other purposes (e.g., estimation of the cochlear size).

The algorithm computes cochleostomy targets starting from the RW and extending inferiorly along the promontory. The cochleostomy target positions match reports of previous histological and clinical studies [12]. Most of the chosen cochleostomy targets (*θ*_*C*_) resulted in a RW related cochleostomy (Figure 5). Using the presented approach, complete ST insertions were accomplished in 9 of 10 cases. In case 1L a scala vestibuli insertion occurred due to an unusually large registration error, which caused an overall drilling error of 0.79 mm. Thus, although the planned trajectory intersected the scala tympani, the drilled position deviated toward the scala vestibuli.

In this study, as compared to previous tests with the robot system, a new self-drilling screw was implemented with the aim of a simpler and more straightforward procedure. The tips of these screws, however, were susceptible to breakage. The localization of the screws in the image data relies on an automatic fitting algorithm based on the shape of the screw. Thus, in cases where the tip of the screw is broken, the algorithm returns an incorrect position. The occurrence of broken screws was present in four samples, but a manual correction of the screw position was able to compensate for the bias in the automatic algorithm. Postoperative evaluation of the registration points revealed localization errors in the range of 0.20–0.50 mm in cases 1R and 1L. Thus, it is very probable that these broken screws were the cause of the high drilling error (0.60 and 0.79 mm) which had not occurred up till now in our collective experience with drilling approximately 30 specimens. Investigations are currently underway to find more robust self-drilling screws which are compatible with our workflow.

Postoperative radiographic assessment showed that the calculated ideal insertion trajectories were effectively aligned with the basal ST. Optimal insertion trajectories passed closely or intersected the facial nerve in all cases. This result closely corroborates those previously reported in a study using microcomputed tomography data of human temporal bones [17]. Therefore, minimization of the angular deviation *δ* of the planned trajectory was mainly restricted by the position of the facial nerve. The average angular insertion depth in this study was observed to be significantly lower as in the previous experiments (319° compared to 606°) [22]. The main difference between the two studies is the fixation method (Sucquet versus Thiel), which is hypothesized to be the major factor that impeded higher insertion depths.

The segmentation of the bony labyrinth represents a crucial step in the presented algorithm. Therefore, errors introduced in this step may have an impact on the computation outcome. One outcome which may occur in case of over segmentation is that the cochleostomy drill would stop short of the endosteum. On the other hand, an under segmentation could possibly cause intracochlear trauma due to a zealous penetration of the cochlea. In this context, the application of additional information gained during the cochleostomy drilling (i.e., force and torque data) could be used to control the drilling depth to stop exactly at the endosteum. Moreover, it is clear that malformations in the basal region of the cochlea (e.g., basal turn ossification) have a strong impact on the computation routines used and are not compatible with the algorithm. Nevertheless, it is assumed that anatomical variations of the RW niche (e.g., an extremely narrow RW) do not influence the computation outcome as long as the RW landmark can be clearly identified [14].

## 5. Conclusions

This study shows that the landmark based approach is a valuable alternative for ST cochleostomy target and insertion trajectory planning in clinical imaging modalities. Although the script utilizes a manual landmark selection and a manual segmentation of the cochlea, targets can be planned in reasonable time (15 min). However, automation of the manual segmentation process is the next step to significantly reduce time. Further, the presented cochleostomy approach is currently being evaluated using perimodiolar electrode arrays.

## Figures and Tables

**Table 1:** Summary of cochleostomy target and drill trajectory planning details.

No.	Distance (mm)					Trajectory alignment (°)		
	FN	ChT	EAC	In/Ma	St	*δ*	*ε*	*θ*_*C*_
1L	0.44	0.12	0.55	2.60	0.65	8	0	12
1R∗	0.37	0.00∗	0.90	2.36	0.62	12	0	10
2L∗	0.37	<0.00∗	0.45	2.64	0.77	11	0	12
2R∗	0.32	<0.00∗	0.45	3.02	0.68	7	0	12
3L	0.37	0.22	1.60	2.78	0.80	12	0	8
3R	0.43	0.53	1.85	2.95	0.65	15	1	4
4L	0.38	1.17	1.89	2.55	0.74	11	1	12
4R	0.38	1.18	2.34	3.11	0.69	12	0	12
5L	0.39	0.37	0.62	3.01	0.51	10	7	4
5R	0.36	0.33	0.90	2.88	0.58	14	1	4

Avg. ± SD	0.38 ± 0.03	0.49 ± 0.45	1.16 ± 0.70	2.79 ± 0.24	0.67 ± 0.09	11.2 ± 2.4	1.0 ± 2.2	9.4 ± 4.1

FN: facial nerve, ChT: chorda tympani, EAC: posterior wall of the external auditory canal, In: incus, Ma: malleus, St: stapes.

*δ*: out of plane alignment between the trajectory and the basal turn plane, Figure 4(a).

*ε*: in-plane alignment of the trajectory in the basal turn plane, Figure 4(b).

*θ*_*C*_: angular position of the cochleostomy, Figure 2(c).

∗Cases with sacrificed chorda tympani because of a small facial recess.

**Table 2:** DCA target accuracy and insertion results.

No.	Target accuracy (mm)	Insertion time (min)	Intracochlear contacts	Angular insertion depth (°)	Implanted scala
1L	0.79∗	5	8 of 12	270	SV
1R	0.60∗	5	10 of 12	330	ST
2L	0.07	5	7 of 12	210	ST
2R	0.15	2	8 of 12	300	ST
3L	0.05	5	12 of 12	420	ST
3R	0.33∗	2	11 of 12	360	ST
4L	0.28	5	8 of 12	290	ST
4R	0.24	7	9 of 12	360	ST
5L	0.27∗	5	11 of 12	300	ST
5R	0.22	4	12 of 12	350	ST

Avg. ± SD	0.30 ± 0.23	5 ± 2	10 ± 2	319 ± 58	

SV: scala vestibule; ST: scala tympani; ∗cases with broken screws which caused varying degrees of error in the registration.
